# Refractive Index Modulation for Metal Electrodeposition-Based Active Smart Window Applications

**DOI:** 10.3390/mi15030334

**Published:** 2024-02-28

**Authors:** Hyojung Kim, Bong Kyun Kang, Cheon Woo Moon

**Affiliations:** 1 Department of Semiconductor Systems Engineering, Sejong University, 209, Neungdong-ro, Gwangjin-gu, Seoul 05006, Republic of Korea; hyojungkim0912@sejong.ac.kr; 2 Department of Electronic Materials, Devices, and Equipment Engineering, Soonchunhyang University, 22, Soonchunhyang-ro, Asan 31538, Chungnam, Republic of Korea; kangbk84@sch.ac.kr; 3 Advanced Energy Research Center, Soonchunhyang University, 22, Soonchunhyang-ro, Asan 31538, Chungnam, Republic of Korea; 4 Department of Display Materials Engineering, Soonchunhyang University, 22, Soonchunhyang-ro, Asan 31538, Chungnam, Republic of Korea

**Keywords:** smart window, refractive index, FDTD, transmission, effective index, nanoparticle, reversible metal electrodeposition

## Abstract

One of the remarkable choices for active smart window technology is adopting a metal active layer via reversible metal electrodeposition (RME). As the metal layer efficiently blocks the solar energy gain, even a hundred-nanometer-thick scale, RME-based smart window has great attention. Recent developments are mainly focused on the various cases of electrolyte components and composition meeting technological standards. As metal nanostructures formed through the RME process involve plasmonic phenomena, advanced analysis, including plasmonic optics, which is beyond Beer–Lambert’s law, should be considered. However, as there is a lack of debates on the plasmonic optics applied to RME smart window technology, as research is mainly conducted through an exhaustive process. In this paper, in order to provide insight into the RME-based smart window development and alleviate the unclear part of plasmonic optics applied to the field, finite-difference time-domain electromagnetic simulations are conducted. In total, two extremely low-quality (Cr) and high-quality (Mg) plasmonic materials based on a nanoparticle array are considered as a metal medium. In addition, optical effects caused by the metal active layer, electrolyte, and nanoparticle embedment are investigated in detail. Overall simulations suggest that the effective refractive index is a decisive factor in the performance of RME-based smart windows.

## 1. Introduction

Nowadays, energy demand is increasing due to technological and industrial advancements, and the demand also leading to climate change on Earth. Numerous approaches have been suggested to mitigate the problem in terms of energy production, saving, and storage. In daily life, energy consumption in housing is important, as there is lighting, heating, and cooling saving issues [1,2]. The control of incident solar energy in a building is a primary requirement to reduce these problems. Low-emissivity glass was considered for energy saving in housing, as it manages solar-heat gain; however, if it is adopted as a standalone solution, it is selectively useful only in one season (summer or winter) and adjustability is not provided [3,4,5]. By reflecting on this perspective, an active switching function is beneficial to glazing. Stimuli-induced optical property modulation, usually thermal and electrical, imparts special functionalities to window frames. Smart window technology has emerged to address the drawback by introducing a switchable functionality [6]. Smart windows are technologically targeted to address both the heating/cooling loss and visual aesthetic improvement. 

The first function of a smart window is a modulation of the primary transmission of solar energy, which refers to the light that emerges inside the housing space through the window. Visual comfort and near-infrared heat gain control functions are achieved. Another function is the modulation of the secondary transmission of solar energy caused by photothermal processes. In thermodynamic equilibrium, the emissivity and absorptivity of thermal radiation are equal, which is known as Kirchhoff’s law of thermal radiation.In reality, a certain proportion of the absorbed light energy is emitted as heat due to various photothermal processes (e.g., plasmonic local heating, non-radiative relaxation, and thermal vibrations) [7,8]. The ratio between the sum of primary and secondary solar energy transmission and total incident solar is called as the solar heat gain coefficient (SHGC). The control of the SHGC according to the weather and region has been a great issue. In addition to that, reflection from the window is also an important issue, as it induces glare phenomena. Therefore, reducing reflection is also a matter for smart window development. From this perspective, an understanding of optical properties is essential in smart window developments, with the related optical wavelength region being considered. The wavelength range of allowed solar light transmission from the Sun to the Earth is called an atmospheric window. The solar radiation spectrum on the ground of Earth is standardized (ASTM G-173) based on air mass 1.5 global (AM 1.5G) condition [9,10]. Solar radiation on the Earth’s ground has its maximum visible (VIS) light region and spans across near-infrared (NIR) region. As there are some exceptions, due to the strong absorption from water vapor in the atmosphere (e.g., 1350–1410 nm, 1830–1930 nm), solar light wavelength range spans from ~300 to ~2500 nm, which is the important optical range for smart window design (also called ‘dynamic window’).

Smart windows that are capable of active modulation have received great attention due to their usefulness in SHGC control. Active functionality allows optical modulation as user-desired, mainly, by adopting electricity. Although liquid crystal-based smart windows are being commercialized, there are some inherent drawbacks. For example, due to the high refractive index difference with the polymer matrix they are embedded in, light is likely to be scattered (haze phenomena). Furthermore, relatively high operation voltage (around 30 to 100 V) is required. Moreover, it is hard to ensure complete transparency or darkness as that type of smart window requires the incorporation of dye molecules to achieve coloration [11,12]. The aforementioned drawbacks could be bypassed by replacing the smart window components with another material. There have been numerous attempts to adopt alternative active layers to smart windows that can change their refractive index through redox mechanisms. Conjugated polymer and transition metal oxides are representative examples. Tinting is performed according to the oxidation state change in conjugated polymer or transition metals by applying a bias [13]. However, due to their low imaginary refractive index value, their light absorption property is relatively weak. As a result, micrometer-sized thicknesses that require higher charge density for the tinting process are required for the fabrication. In addition, since these materials possess a bandgap, it is likely to exhibit colors, which result in a color neutrality deviation in the tint or transparent states. To overcome these drawbacks, the metal active layer is adopted via the reversible metal electrodeposition (RME) process in smart window technology.

RME-based active smart windows, which are operated by electricity, are composed of a transparent and conductive working electrode (usually, indium tin oxide; ITO), a counter electrode (usually, metal) that enables enough current to flow occurring in the reaction, an electrolyte to provide an electrical connection between the two electrodes, and an inert edge sealant to contain and seal the electrolyte. Metal ions and other compensating ions are dissolved in the electrolyte to provide RME reaction, electrical conductivity, and shape control of the electrodeposits. An RME smart window operates tinting by precipitating metal ions dissolved in the electrolyte on the transparent conductive working electrode through an electrochemical reduction reaction by forming the metallic film. The transparency state is achieved again by dissolving the metallic, thin film into the electrolyte through an electrochemical oxidation reaction. Metallic electrodeposited film is composed of metal and its complex composites. Therefore, the optical property of metal should be considered due to its significant effect on tinting. Interestingly, the nanostructure of the metal electrodeposits can be variously tuned through the composition of ions in the electrolyte and the biasing methods. 

To provide sufficient light-blocking properties to the smart window, the electro-deposit should possess a high light attenuation. According to the unity relation between transmission, reflection, and absorption, the sum of reflection and absorption must be increased after tinting. However, an increase in reflection is not preferred in the application, as it induces high glare to the surrounding area. Absorption is very essential as it is related to the emissivity characteristics in the infrared range, secondary solar energy transmission due to the photothermal effect, and black color realization during tinting. Overall, the development of RME-based smart windows should consider the following: (1) increase the portion of absorption, (2) high color neutrality, and (3) high transparency without haze in the transparent state. Note that, if there is a dense structure that does not possess effective nano-sized morphology, a strong reflection and bulk coloration are expected due to the rise of bulk property. The above three criteria cannot be achieved from bulk-like film. The particles resulting from electrodeposition should be formed so as not to exhibit bulk characteristics. Therefore, chemical species, morphological modifications, and biasing are very important for the formation of nanostructures. As previously shown in the Ag-based electrochromic device suggested by the Kobayashi group, the shape of the thin film (decoration with ITO particles) and the ion combination of electrolytes have a strong effect on absorption [14]. Note that, as Ag possesses a strong localized surface plasmon resonance (LSPR) in the visible light region, color neutrality is high (likely to have a color). In this view, the selection of metal ions highly affects not only the optical properties but also the tinting energy efficiency of smart windows. 

There are two key optical factors for the selection of smart window metals. The first is the imaginary part of the refractive index (*k*), as it is proportional to the absorption coefficient; the larger its value, the greater the absorption of light. The second is plasmon resonance. When the electromagnetic wave hits the dielectric/metal interface, resonance between electrons at the metal surface and the incident electric field occurs, which is called plasmon resonance. Plasmon resonance endows a unique optical property to metal nanostructures, different from that of bulk-sized metals, and complicates the prediction of optical characteristics that would otherwise be straightforward under Beer–Lambert’s law. Plasmon resonance causes enhanced light absorption. Therefore, plasmon resonance phenomena from independent metal nanostructures (electrodeposits) on dielectric (transparent conducting substrate) could be categorized into two major types. Resonance between the dielectric/metal interface is called surface plasmon polariton (SPP), which results in a strong electric field on the dielectric part. Another phenomenon is LSPR, which occurs in the nano-sized structure due to the quantum confinement effect. LSPR phenomena lead to a high electric field intensity at the metal surface, parallel to the direction of the electric field oscillation direction. LSPR causes strong absorption around specific wavelength ranges. Well-known examples of these metals include Au, Ag, and Cu nanoparticles, as they possess resonance within the visible light region. For these reasons, plasmon resonance plays an important role in the optical properties of electrodeposits. 

Herein, to provide an optical interpretation basis and insight into the further RME smart window development, finite-difference time-domain (FDTD) electromagnetic simulations of the metal nanostructure and its components in the RME smart window are performed in terms of refractive index modulation. Nanomaterials with relatively low-quality (Cr) and high-quality (Mg) plasmonic properties were selected as two extreme examples that were suggested in previous research [15]. Advantages in transmission with metal electrodeposits are investigated via hypothetical metal nanoparticle array simulations. A lower transmission compared to the equivalent mass of thin flat film is observed in some visible light regions with nanoparticle array. The origin of abnormal low transmission is pointed out by an electric field component analysis, which shows a strong SPP resonance in high-quality metal. As plasmonic property is highly dependent on the refractive index of the surrounding medium, other simulations are performed by changing the refractive index of the electrolyte to provide the criteria for the electrolyte selection problem. Lastly, transmission enhancement is explained with effective refractive index calculations.

## 2. Optical Simulations

The refractive index of ITO is adopted from the literature [16]. The refractive index of Mg is adopted from Hagemann et al. [17]. The refractive indexes of SiO_2_, Cr, and H_2_O are adopted from Palik [18]. Electromagnetic simulations are proceeded with a finite-difference time-domain (FDTD) program (Lumerical Solutions, v.2024 R1). A simulation region of 40 × 40 × 3500 nm^3^ size is constructed. Periodic boundary conditions are provided in the *x* and *y* direction. A perfectly matched layer (PML) boundary condition is provided in the *z*-direction. A plane wave source light is imported into the entire simulation. The p-polarization case is only considered due to the symmetry of the simulated nanostructure. A mesh volume of 0.5 × 0.5 × 0.5 nm^3^ is used throughout the entire simulation.

## 3. Results

**Figure 1:**
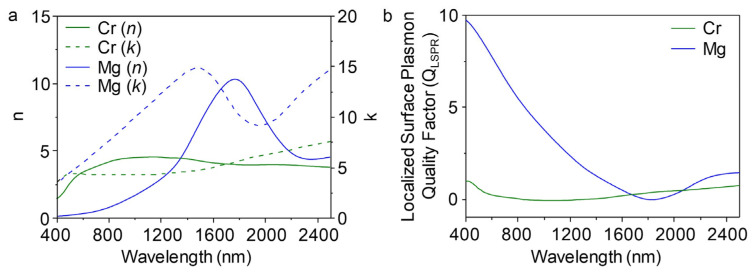
The refractive index of two representative metals. (**a**) Complex refractive index of Cr and Mg. (**b**) Plasmon quality factor of Cr and Mg.

In our previous research, near-field enhancement properties of various metals were discussed using the plasmon quality factor (Q) at a wavelength of 500 nm [15]. To delve into the discussion, two metal materials, chromium (Cr) and magnesium (Mg), were selected. The real part of the complex refractive index was represented as ‘*n*’, and the imaginary part of the complex refractive index was represented as ‘*k*’. As shown in Figure 1a, metals possess higher values of *k* from the VIS to the NIR range compared to the other electrochromic materials. For example, oxides commonly used for electrochromic applications, like WO_3_, have an effectively zero *k* value in their intrinsic state [19,20]. While it could have some extent value with Li intercalation, it is yet to be small compared to the metals [21,22,23]. Metal’s high value of *k* brings a clear advantage when it is in the application of a smart window active layer. Since the *k* value is proportional to the optical absorption coefficient, a higher *k* value results in a stronger light absorption. According to the refractive index data, Mg possesses a higher *k* value than Cr, which suggests Mg could absorb more solar energy than Cr. Complex refractive indices allow the calculation of complex permittivity, which is used to yield plasmon quality factors. The real part ($Re{(\varepsilon }_{M})$) and imaginary part ($Im{(\varepsilon }_{M})$) of the metal’s dielectric function are expressed as follows:

(1) $$Re{(\varepsilon }_{M})=n^{2}-k^{2},\;Im{(\varepsilon }_{M})=2nk.$$

To describe the performance of the plasmonic system, the plasmon quality factor (ratio of enhanced local field and incident field) is introduced. The value is yielded from complex permittivity. As there are two types of plasmon resonance (SPP and LSPR), two types of quality factors could be defined. For the spherical nanoparticle, quality factors are defined as $Q_{SPP}=\frac{({Re{(\varepsilon }_{M}))}^{2}}{Im{(\varepsilon }_{M})}$ (for SPP) and $Q_{LSPR}=-\frac{Re{(\varepsilon }_{M})}{Im{(\varepsilon }_{M})}$ (for LSPR) [24,25,26]. Although these definitions are slightly different, both values have similar implications. Since the optical simulation is based on a nanoparticle array, the quality factor definition of LSPR is used as the representative value. Figure 1b shows the calculated Q_LSPR_ values. These numbers describe how much the local field is ideally enhanced compared to the incident field. Therefore, a high Q_LSPR_ value indicates the capability of creating strong absorption in the wavelength region through strong plasmonic oscillations. For Mg, the Q_LSPR_ tends to increase more when it comes to approaching shorter-wavelength regions. When Mg is considered an active layer for smart window application, the high-quality factor of Mg indicates that, especially, in the VIS region, the light wave is likely to attenuate notably more compared to the Cr. By reflecting on this view, nanoparticle array simulations are performed to observe the optical characteristics in the nanostructure. 

**Scheme 1:**
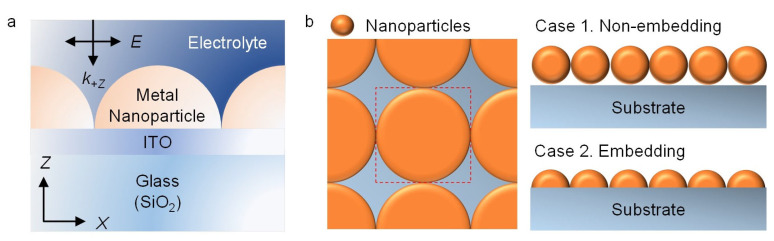
Simulation configuration of nanoparticle array. (**a**) Simulation configuration. (**b**) Arrangement of particles.

The basic structure of the simulation array to be discussed later is depicted above in Scheme 1. The refractive index information of the adopted materials is commented on in the experimental section. A 130-nm-thick layer of ITO is located on top of a SiO_2_ glass. Nanoparticles (in the figure, the size of the nanoparticles is somewhat exaggerated for clarity) are formed over the ITO layer. The electric field oscillation direction of incident light is set parallel to the *x*-axis (p-polarized light). If the light propagates from the electrolyte side to the glass side, it is defined as an outdoor illumination using the wavevector (*k* + Z). Similarly, if the light propagates from the glass side to the electrolyte side, it is defined as indoor illumination using the wavevector (*k* − Z). This is depicted in Scheme 1a. Simulations are performed with outdoor illumination if there is no comment. Metal particles over the ITO form a square array, and it is distinguishable by two major scenarios according to the contact state (Scheme 1b). When the size of the nanoparticle decreases, the nanoparticle array converges into a thin film, which loses interparticle optical interactions. Vice versa, when the size of nanoparticles increases, the system converges into an ITO-only case, except for Mie resonance in the very narrow wavelength range [15,27]. Therefore, a 40 nm diameter is adopted in the following simulations. By assuming a non-contact configuration with ITO, the spherical-shaped particles are considered (Case 1). Conversely, by assuming a major contact configuration with ITO, the hemispherical-shaped particles are considered (Case 2). When particles are formed through electrodeposition, it can be considered an intermediate case between Cases 1 and 2 [28]. The simulations in the next sections consider both cases. Due to the polarization dependency of the hexagonal array, it is not considered for the simulation. Note that discussions for nanoparticle multilayer are not conducted here; Readers could refer instead to our previous research [15]. In short, transmission eventually reaches zero by increasing the number of layers, but tinting efficiency is dependent on the morphology and contents. For reference, the amount of charge ratio between Mg and Cr that is required to make the same thickness from electrodeposition is approximately 1:2.885 [29]. 

**Figure 2:**
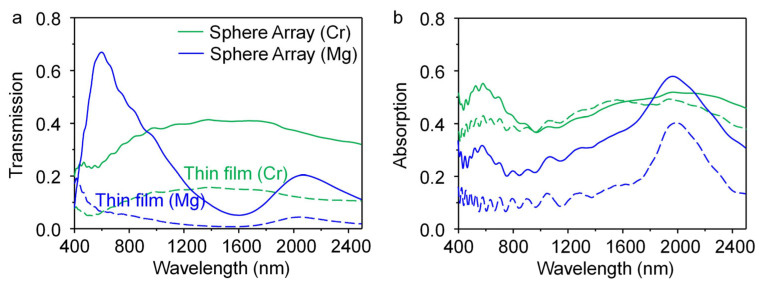
The simulated optical property of sphere particles square array with outdoor illumination. The green color indicates Electrolyte/Cr layer/ITO/SiO_2_’s optical simulation data, and the blue color indicates Electrolyte/Mg layer/ITO/SiO_2_’s optical simulation data. Sphere array optical data are indicated as solid lines. Thin film optical data equivalent mass with sphere array are indicated as the dotted lines. (**a**) Transmission and (**b**) absorption is shown in the range of 400–2500 nm.

Optical properties from sphere arrays are investigated in detail with the outdoor illumination simulation configuration. A square array of spherical nanoparticles with a diameter of 40 nm is compared with a thin film of the equivalent molar mass. An optical characteristic comparison with an equivalent molar mass has significant implications, as it counts the amount of charge that would require making target optical transmission. If a structure achieves a greater transmission attenuation with a smaller amount of charge, that means more efficient tinting is possible. To figure out the primary transmission attenuation efficiency, the transmission properties are examined. As seen in Figure 2a, in the case of Cr, the thin film outperformed the nanoparticles in light blocking across all wavelength ranges. This result can be understood as the sum of the two interactions, the decrease in transmission due to plasmon resonance, and the transmission increase due to the generation of metal-free areas. Since Cr possesses a low-quality plasmon resonance factor, the absorption enhancement due to plasmon resonance should not be strong. So, transmission attenuation from plasmon resonance would be weak. The case of Cr indicates that the transmission increase in the metal-free area significantly influences the optical properties. In contrast, Mg is a metal with a high plasmon quality factor, exhibiting a transmission profile significantly different from Cr. Most importantly, in contrast to Cr, which mostly did not surpass the thin film in reducing transmission, there is some region in the short wavelength where the transmission from the nanoparticle array is lower than that of the thin film. This inspiring result shows that the selection of metal plays an important role in transmission attenuation due to the different plasmonic abilities. However, it should be noted that as these results are spherical-particle based, a detailed investigation will be given with hemispherical particles in the following section. 

The meaning of transmission is more clarified by examining absorption. In Figure 2b, absorption profiles of spherical nanoparticles with a square array are displayed. Absorption of the equivalent mass of thin films is also attached for reference. Oscillations come from multilayer interference (Fabry–Pérot interference) [30,31]. Interestingly, while there are some regions where Cr shows an enhanced absorption compared to the thin film, the absorption enhancement is not higher compared to the Mg. By examining the plasmon quality factor profile in Figure 1b, relevance with the absorption profile could be found. The minimum point of absorption enhancement (the ratio of absorption between the sphere array and thin film) is analogous to the plasmonic quality factor profile (Figure S1). This suggests that absorption enhancement is caused by plasmon resonance. The absorption enhancement is relatively low, as Cr possesses a weak plasmon resonance. In contrast, for Mg, it is noticeable that the absorption enhancement is considerably higher across all regions compared to the thin film case. An interesting property is that, compared to the thin films, the nanoparticle arrays always reduce reflectance (as shown in Figure S2). In contrast with the flat-film layer, the reflected energy from the metal nanoparticles is re-interacted with other metal nanoparticles which results in enhanced absorption. From the Mg spherical particle array case, almost zero reflection is exhibited around the 600 nm wavelength range and reflection minimum around 2000 nm. At that range, there is an increase in absorption which could be found as a peak. In summary, as a result of plasmon resonance from particles, reduced transmission dips are formed [32]. 

**Figure 3:**
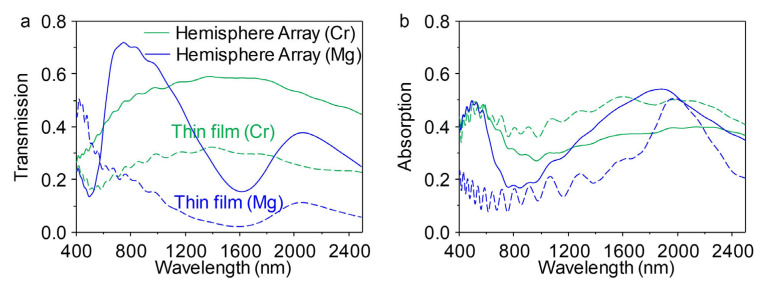
The simulated optical property of hemisphere particles with a square array form with outdoor illumination. The green color indicates Electrolyte/Cr layer/ITO/SiO_2_′s optical simulation data and the blue color indicates Electrolyte/Mg layer/ITO/SiO_2′_s optical simulation data. Sphere array optical data are indicated as solid lines. Thin film optical data equivalent mass with sphere array are indicated as the dotted lines. (**a**) Transmission and (**b**) absorption is shown in the range of 400–2500 nm.

Another optical simulation with hemispherical particles arranged within a square array is conducted with outdoor illumination (Figure 3). The shape difference ensures an aerial interface formation with the dielectric substrate (ITO) and particles. In the previous simulation (Figure 2), the metal–dielectric interface is not formed, so there is no optical contribution from the interfacial effect. For reference, a thin film corresponding to the same molar mass with a particle array is compared. When there is a metal–dielectric interface, in addition to the plasmon resonance of independent particles (LSPR), another resonance based on SPP is emerging. Therefore, a different optical characteristic is expected compared to the optical data from Figure 2. As seen in Figure 3a, in the case of Cr, the hemispherical array also does not exhibit an enhancement of transmission attenuation compared to the thin film. Even the absorption is below the thin-film case. This implies in the transmission that the existence of a metal–dielectric interface has no positive effect on the attenuation, as Cr possesses a low-quality plasmon resonance. However, Mg exhibits a completely different story. In the transmission profile of Mg, the hemispherical particle array shows a wide range of transmission reduction compared to the thin film in the range from around 400 to 600 nm. Remarkably, this transmission reduction is expressed in a wider range than when simulations were conducted with spherical nanoparticles. This is attributed to the SPP resonance caused by an optical interaction at the Mg metal and ITO substrate interface. This phenomenon will be investigated in detail in the next section by analyzing the field profiles. From the hemispherical Mg particle array, two transmission minima are observed. When compared to the data in Figure 2a, while the position of the longer wavelength minimum remains the same (around 1600 nm), the position of the shorter wavelength dip shifts to the longer wavelength. This indicates the presence of a different form of plasmonic interaction in this region. Note that, transmission dip shift is not observed in the case of Cr.

By observing the absorption profile, intriguing features can be seen, as shown in Figure 3b. By referring to the example in the previous section, there are certain ranges of increased absorption with a spherical Cr particle. However, with the use of hemispherical Cr particles, no increase in absorption is observed. This indicates that forming a metal–substrate interface using low-quality plasmonic metal is not beneficial to light attenuation. On the other hand, with hemispherical Mg particles, an increase in absorption across all ranges compared to the thin film is observed. This result demonstrates that high-quality plasmonic materials, when forming an interface with the dielectric, materials act as strong attenuators to the light. Note that, absorption peaks would be broader if randomness is included [33]. The absorption enhancement factor (the ratio of absorption between the sphere array and thin film) is also achievable with absorption data. By comparing with the absorption enhancement factor in Figure S1, the positions of the minima and shape are similar (Figure S3). This suggests that the plasmonic quality factor is the major determinant of these minimum values of absorption enhancement, and light attenuation ability depends on the structural factors. The above simulations could be an essential criterion for the active layer selection of smart windows. By examining reflectance, reflectance is reduced compared to the thin-film materials, as shown in Figure S4. The energy of the reflected light metal surface could re-interact with the surface of other metals, which results in a reflection reduction. Though the dip of reflectance values coincides with the absorption peaks in the spherical particle example, in this case, these are not necessarily coinciding. For instance, in the case of Mg, two absorption peaks can be observed around ~500 nm and ~1900 nm, whereas the reflection dips are located at ~700 nm and ~2000 nm. Particularly around the 400–600 nm range, both absorption and reflection have peak values together. These unique phenomena are attributed to the light interaction with the metal–substrate interface rather than interparticle resonance.

**Figure 4:**
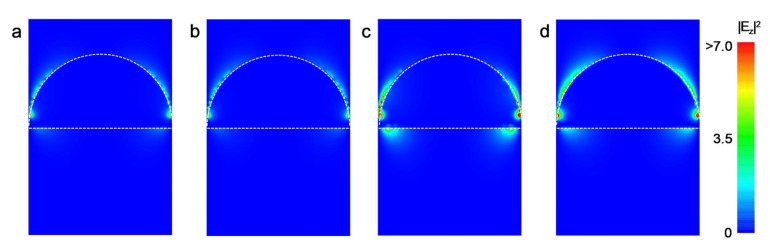
*X* (horizontal)–*Z* (vertical) cross-sectional electric field profiles of unit cells from hemispherical nanoparticle array with outdoor illumination. A cross-sectional 40 × 60 nm^2^ area is selected to display the field profile. Electric field intensity parallel to the *Z* direction (|E_Z_|^2^) is depicted. The boundary of the electrolyte, hemispherical particle, and ITO substrate are shown as white-dotted lines. Cr hemispherical particle at (**a**) 500 nm and (**b**) 1000 nm incident wavelength. Mg hemispherical particle at (**c**) 500 nm and (**d**) 1000 nm incident wavelength.

A key indicator of plasmon resonance in nanostructure is an electric field component parallel to the direction of light propagation. The corresponding component is denoted as E_Z_. As the intensity is proportional to the square value of the electric field, the field is displayed with |E_Z_|^2^. In conventional thin films, such a component typically does not arise. Looking at the electric field profile of the Cr hemispherical particles (Figure 4a,b), regardless of the wavelength, the electric field is most strongly focused on the circular boundary between the particle and the electrolyte. The region is also corresponding to the gap space between particles, implying focused electric fields through the interparticle gap. The presence of such resonance strongly supports the LSPR-based absorption mechanism. In contrast to that, the electric field component does not prevail at the particle–ITO interface. This indicates that an SPP resonance-based absorption mechanism cannot be expected at the interface between the Cr hemispherical particles and ITO. In terms of absorption, the attenuation of light in hemispherical particles was less than that of thin films, as seen in Figure 3b. This result can be understood from a relatively weak LSPR resonance phenomena in the Cr system. Comparing the intensity of the electric field at the Cr hemispherical particle–electrolyte interface with the Mg hemispherical particle–electrolyte interface, it is evident that the plasmon resonance on the surface is stronger in Mg than in Cr. 

As expressed in Figure 4c,d, a relatively strong electric field component (E_Z_) is observed at the particle–ITO interface, suggesting the occurrence of a strong SPP resonance. Such a kind of resonance is not evident in the previous example with Cr. In the absorption spectrum with hemispherical Mg particles (Figure 3b), the 500 nm is around a peak region, and the 1000 nm region is around a dip region. The field profile accompanies high |E_Z_| field intensity corresponding to absorption profiles, and resonance shows higher in the high absorption peak region rather than dip region. Especially, in the 500 nm region, where both absorption and reflection simultaneously increase, the E_Z_ field profile possesses a strong SPP resonance beneath a hemispherical Mg particle. Due to the occurrence of SPP resonance, the E_Z_ component concentrates at the interface between the flat ITO and Mg hemispherical particle which enhances reflection and light attenuation. This effect is a unique property that is not observed in other wavelength regions. Overall analysis indicates that while Cr seems to absorb light only at the particle–electrolyte interface, Mg appears to absorb light both at the particle–electrolyte interface and the particle–ITO interface. A field component analysis clarifies the absorption characteristic results in Figure 3b and rationalizes an enhanced absorption compared to the thin film using Mg.

**Figure 5:**
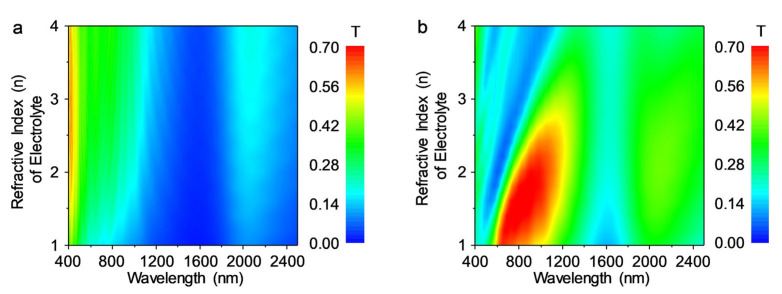
Refractive index tuning of electrolyte (**a**) with 10.5 nm Mg thin-flat layer and (**b**) with hemispherical Mg particle square array (diameter of 40 nm). FDTD simulation configuration is the same as Scheme 1 and outdoor illumination is adopted. Transmission is displayed.

To extend the above understanding to the electrolyte system, FDTD simulations are conducted by varying the refractive index of an ideal (non-absorbing) electrolyte using the Mg-based system as an active layer. Figure 5a presents a transmission calculated by adopting an Mg thin-flat layer by changing the refractive index from one to four. It is shown that the transmission reaches local maxima near the 400 nm wavelength region, local minima near the 1600 nm wavelength region, and local maxima near approximately the 2100 nm region. This repeated peak and dip is a characteristic trend known in the multilayer film theory [34]. The positions of these peaks and dips are calculated to be fixed, and calculation is possible using analytic methods like transfer matrix or FDTD methods. Thin-flat film-based optical phenomena could be distinguishable from the above approach. 

In contrast, Figure 5b shows the results of transmission mapping from a square array of hemispherical Mg particles, by varying refractive indices of the electrolyte. In thin-film structures, transmission has a peak around 400 nm, and it increases by approaching shorter visible wavelength regions, which is a disadvantage for smart window applications. This issue can be resolved by adopting nanostructures that have plasmonic properties because the plasmon resonance introduces a new absorption mechanism that induces increased attenuation. It is theoretically known that the resonance wavelengths of LSPR and SPP vary depending on the refractive index [35]. In the simulation, as the refractive index increases, a redshift can be observed in the transmission dip and peak bands. The sensitivity of the refractive index to the plasmon resonance is exemplary and is shown through transmission mapping. An interesting observation is that the characteristics of the transmission peaks and dips in the thin film persist. For example, the transmission dip at around 1600 nm is still present, unaffected by the refractive index modulation. This implies that overall optical property is determined by the sum of thin-film properties and nanostructure-induced plasmonic properties. In this example, it was observed that as the refractive index increases in the Mg hemispherical square array, especially in the visible light range, the transmission tends to be lower than that of the thin film. These simulations indicate that, when designing active layer materials for smart windows, the refractive index should be considered, and both the elements of thin films and nanostructures should be taken into account.

**Figure 6:**
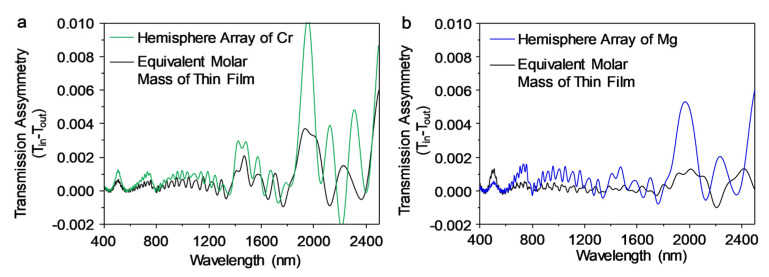
Transmission asymmetry (defined as a difference of transmission from indoor illumination (*k* − Z) and transmission from outdoor illumination (*k* + Z)) thin-flat film and a hemispherical square array of (**a**) Cr and (**b**) Mg.

To examine the transmission asymmetry in terms of a light propagation direction, a comparison is made between the transmission values under indoor illumination and outdoor illumination. The reason for this asymmetry is due to the non-symmetrical placement of the active layer, which is skewed to one side. Especially, when plasmonic nanostructures are present, dipoles or quadrupoles can be generated, resulting in a directional dependence of the effective refractive index [36,37]. This asymmetry is crucial in smart windows as it imparts directional selectivity to light transmission, making it a key factor to consider in actual designs accounting for summer and winter conditions. As seen in Figure 6, hemispherical nanoparticles exhibit an increased asymmetry in transmission compared to thin-flat films. However, it is worth noting that this difference yields around 1% in the simulated nanostructures, which are only considered a hemispherical particle single layer and do not account for three-dimensional structures. In actual devices using thicker films, as is not reported yet, greater asymmetry in transmission would exist as observed in simulations. As shown in Figure 6a, the introduction of hemispherical particles using Cr increases the asymmetry in transmission. Similarly, as indicated in Figure 6b, an increase in transmission asymmetry is observed in most regions. However, in the 400–600 nm range, where SPP phenomena are dominant, a tendency for decreased transmission asymmetry is observed. This could be a criterion when designing actual devices using high-quality plasmonic materials where SPP phenomena dominant the wavelength region. 

**Figure 7:**
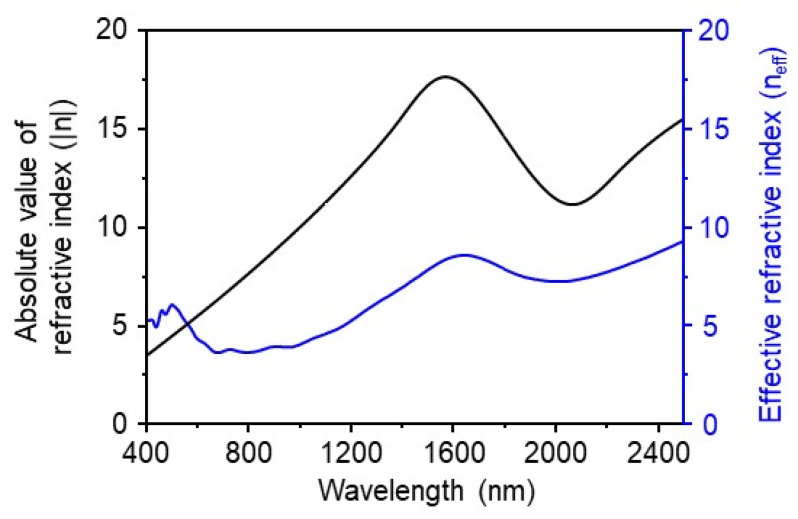
Comparison of the absolute value of the refractive index (|n|) of Mg with effective refractive index (n_eff_) of Mg hemispherical particle square array. The effective refractive index is calculated by assuming a 20-nm-thick ideal dielectric medium on top of ITO. Outdoor illumination is used.

To figure out the interrelationship between the refractive index and transmittance, the effective index of the Mg hemispherical particle square array is calculated. As classical approximations like the Maxwell-Garnett or Bruggeman theory are used to calculate the mixture refractive index of metal, it does not need to be analogous with the effective index of metal nanostructure, due to the occurrence of evanescent field-originated phenomena such as plasmon resonance [38,39]. The effective refractive index is calculated by assuming the effective thickness of the thin-film medium layer as 20 nm, which is the radius of the hemispherical particles. An ideal lossless dielectric medium that only has a real refractive index is adopted for the calculation. The direction of light propagation is set as outdoor illumination. The refractive index of the corresponding structure is shown in Figure 7. In most wavelength regions, the absolute value of the refractive index of Mg material is greater than its effective refractive index from the hemispherical particle square array. Considering a water electrolyte having a real part of the refractive index of approximately 1.33–1.26 from 400 to 2500 nm, which fills the interparticle space, it can be expected that the effective dielectric constant would generally be lower. In the simulation, it was observed that from around 400 to 600 nm regions, the light attenuation of the nanostructures is greater than that of the thin films. This result indicates that increasing the magnitude of the effective refractive index in nanostructures is key to enhancing absorption.

## 4. Conclusions

To provide design principles of optical properties in metal electrodeposition-based smart window systems, various combinations are investigated in terms of the refractive index. The meaning of the plasmonic quality factor, the refractive index of the metal and electrolyte, and the effective refractive index by nanostructures in the RME smart windows are thoroughly examined using electromagnetic simulations (FDTD). Beyond the selection of high-quality plasmonic material, the overall investigation indicates that the effective refractive index should be higher than the metal’s original refractive index and the use of a high-refractive index electrolyte can be an effective tinting mechanism in RME smart windows. The simulation suggests that LSPR phenomena from nanostructuring and the SPP by the presence of a dielectric interface result in enhanced light attenuation. Through the electric field mapping, plasmonic effects were discernible, and the examination of the effective refractive index allowed the optical range of high attenuation. Further in-depth research into more complex multilayer structures or various nanostructures is necessarily required for the realization of high-performance RME-based smart windows.

## Data Availability

Data are contained within the article.

## Supplementary Materials

The following supporting information can be downloaded at: https://www.mdpi.com/article/10.3390/mi15030334/s1, Figure S1: Absorption enhancement factor (sphere particles); Figure S2: Reflection (sphere particles); Figure S3: Absorption enhancement factor (hemisphere particles); Figure S4: Reflection (hemisphere particles).
