# Supplementary material for: Refractive Index Modulation for Metal Electrodeposition-Based Active Smart Window Applications
Source: Micromachines (Basel). 2024 Feb 28;15(3):334. doi: 10.3390/mi15030334 (PMC10972178; doi:10.3390/mi15030334)
Supplement: Supplementary file 1 [file micromachines-15-00334-s001.zip › micromachines-2886799-supplementary.pdf]

Supplementary Information

Refractive Index Modulation for Metal Electrodeposition-based Active Smart Window Applications

Hyojung Kim,[1] Bong Kyun Kang,[2] and Cheon Woo Moon[2,*]

1 Department of Semiconductor Systems Engineering, Sejong University, 209, Neungdong-ro, Gwangjin-gu, Seoul, 05006, Republic of Korea

2 Department of Display Materials Engineering, Soonchunhyang University, 22, Soonchunhyang-ro, Asan-si, Chungnam, 31538, Republic of Korea;

[*] Correspondence: cwmoon0810@gmail.com

[†] Both authors contributed equally to this work.

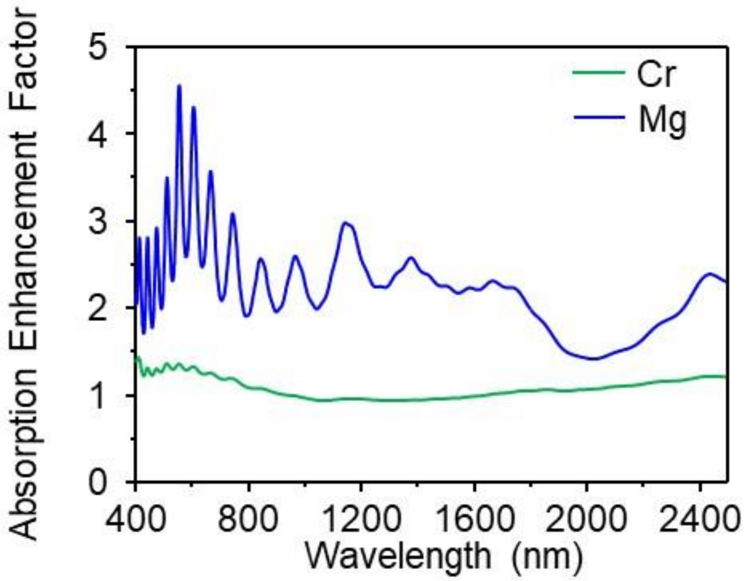

Figure S1. Absorption enhancement factor (ratio of absorption from sphere particles square array and thin film) from outdoor illumination. Electrolyte/Cr layer/ITO/SiO_2 and electrolyte/Cr layer/ITO/SiO_2 are considered.

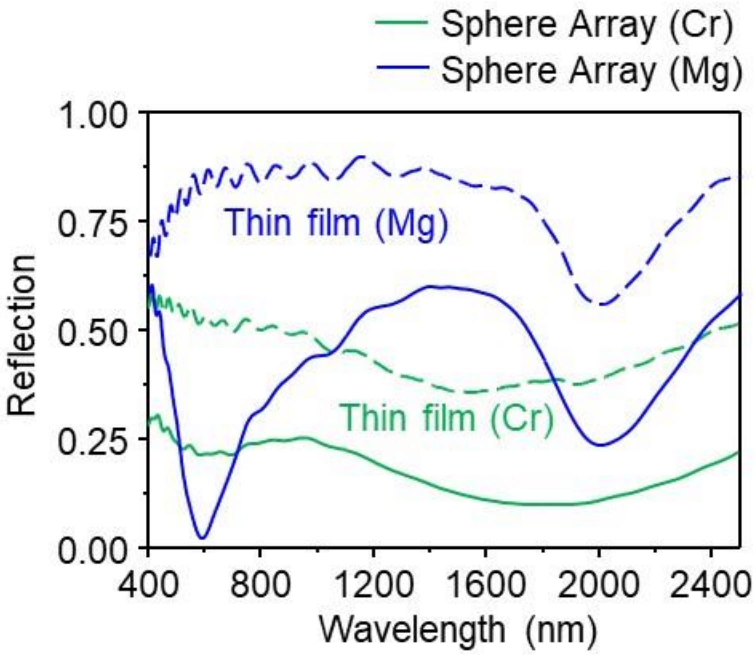

Figure S2. Simulated reflection of sphere particles with square array form outdoor illumination. Thin film optical data equivalent mass with sphere array is indicated as a dotted line.

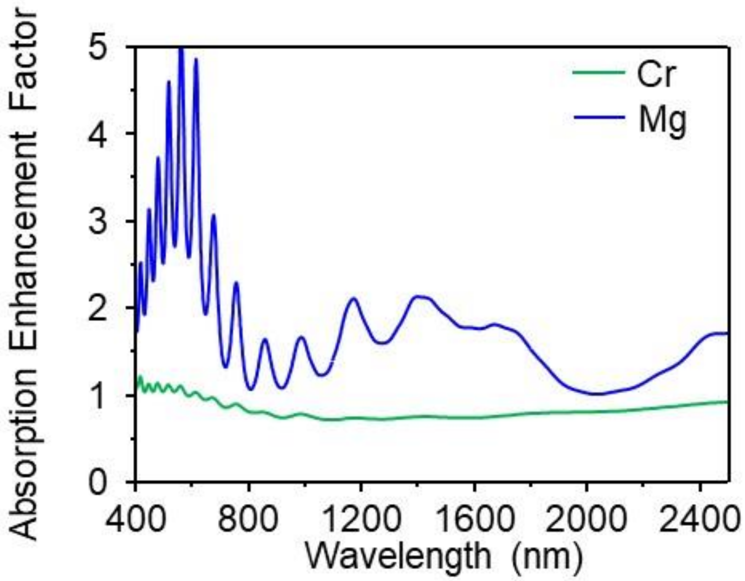

Figure S3. Absorption enhancement factor (ratio of absorption from hemisphere particles square array and thin film) from outdoor illumination. Electrolyte/Cr layer/ITO/SiO_2 and electrolyte/Cr layer/ITO/SiO_2 are considered.

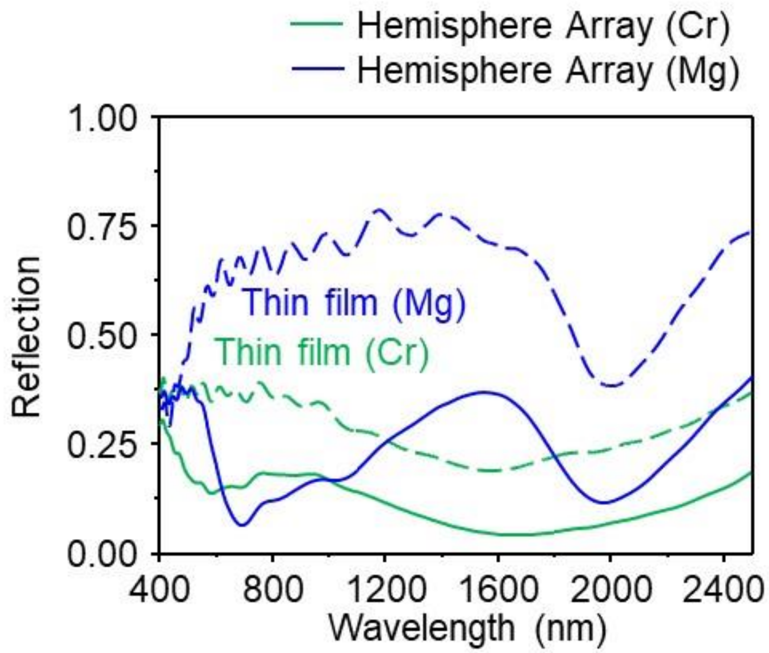

Figure S4. Simulated reflection of hemisphere particles with square array form outdoor illumination. Thin film optical data equivalent mass with hemisphere array is indicated as a dotted line.
